# Needed adaptations in psychological treatments for people with vision impairment: A Delphi study, including clients, relatives, and professionals

**DOI:** 10.3389/fpsyg.2023.1028084

**Published:** 2023-01-30

**Authors:** Jessica Braakman, Paula Sophia Sterkenburg

**Affiliations:** ^1^Department of Clinical Child and Family Studies and Amsterdam Public Health Research Institute, Vrije Universiteit Amsterdam, Amsterdam, Netherlands; ^2^Acadamic Lab ‘Social relations and attachment’ Bartiméus, Ons Tweede Thuis, Vrije Universiteit Amsterdam, Amsterdam, Netherlands; ^3^Bartiméus, Oude Arnhemse Bovenweg 3, Doorn, Netherlands

**Keywords:** visual impairment, psychological treatment, adaptations, expert experience, Delphi study

## Abstract

**Objective:**

In this study, we aimed to identify the themes that should be addressed when adapting mental health treatments for adults with a visual impairment.

**Method:**

A Delphi study was conducted among 37 experts, including professionals, persons with a visual impairment, and relatives of clients with a visual impairment.

**Results:**

The Delphi consultation revealed seven categories (factors) that were identified as important in the treatment of mental health problems for clients with a visual impairment: the visual impairment, environment, stressors, emotions, the professional’s role and attitude, treatment setting, and accessibility of materials. Factors regarding the clients’ visual impairment, such as the severity of the impairment, influence the extent to which adjustments are needed in treatment. During treatment, the professional plays an important role in explaining any visual elements that a client with a visual impairment may miss.

**Conclusion:**

In psychological treatment, clients require individual adaptations for their specific visual impairment.

## 1. Introduction

According to the World Health Organization (WHO), 2.2 million people worldwide suffer from vision impairment (World Health Organization, 2019), which includes both blindness and low vision, and implies the partial or complete inability to see, due to congenital condition, disease, or injury (World Health Organization, 2019). It is estimated that 2,50,000 people in the Netherlands have a visual impairment, of whom 85% are 50 years or older (Oogfonds, 2021).

Visual impairment is associated with reduced quality of life, increased illness, and mental fatigue and can induce feelings of social isolation and loneliness (Chia et al., 2004; Mojon-Azzi et al., 2008; Limburg and Keunen, 2009; Fenwick et al., 2012; Kempen et al., 2012; van der Aa et al., 2016). Moreover, people with vision impairment may be at greater risk of developing psychological problems, such as depression and anxiety (Evans et al., 2007; van der Aa et al., 2015, 2016; Demmin and Silverstein, 2020; van Munster et al., 2021). Among the general global population, 4.4% of individuals suffer from depression and 3.6% from anxiety disorder (World Health Organization, 2017, 2019). In contrast, among people with a visual impairment, one-third experience depression and/or anxiety. Depression and anxiety can have severe consequences, potentially leading to increased illness, suicide, and mortality (Reynolds and Kupfer, 1999; Rees et al., 2010; van der Aa et al., 2016; Demmin and Silverstein, 2020). In addition, compared to people without visual impairment, people with a visual impairment are at a higher risk of certain traumatic events—such as falls, traffic accidents, and abuse. These traumatic events may lead to a variety of stress reactions and mental health problems (McFarlane, 1988; Au-Yong and Firth, 2006; Saur et al., 2017; Brunes et al., 2019; van der Ham et al., 2021).

Although several studies demonstrate that people with a visual impairment exhibit an elevated prevalence of psychological problems—including depression, anxiety, and suicide—these problems remain largely untreated (Burmedi et al., 2002; Hayman et al., 2007; Nijman et al., 2010; Bookwala and Lawson, 2011; Holloway et al., 2013; Senra et al., 2016; Demmin and Silverstein, 2020). For example, Rees et al. (2010) found that among 143 adults with a visual impairment, 60 (42.0%) were diagnosed as depressed. However, no one in that study sample was receiving treatment for depression at the time of the study (Rees et al., 2010; Senra et al., 2016; Demmin and Silverstein, 2020). Multiple psychological and psychosocial interventions are regarded as evidence-based treatment options for various psychological problems in the general population (Hunot et al., 2007; Cuijpers et al., 2008; Barth et al., 2013; Connolly Gibbons et al., 2016). However, recent studies highlight the need for tailored interventions to treat psychological problems in people with a visual impairment (van der Aa et al., 2015; Senra et al., 2016). It may be necessary to adjust interventions for people with a visual impairment. In the present study, we aimed to identify the themes that should be addressed in adapting treatments. The research question was as follows: *Which adaptations are needed in psychological treatments for adults with a visual impairment?*

## 2. Materials and methods

### 2.1. Design

We conducted a qualitative study in which the Delphi method (Keeney et al., 2011) was used to establish a consensus of experts—including experts by experience and their relatives, as well as professionals who work with clients with a visual impairment—regarding the adjustments required in psychological treatments for people with a visual impairment (Keeney et al., 2011) The Delphi study comprised two rounds (Keeney et al., 2011): First, face-to-face interviews were conducted with participants. In the second round, an online survey tool was used to evaluate and rank the themes (concepts) identified in the first round. The third round was not needed because consensus on the important topics was reached in round 2.

### 2.2. Participants

The Delphi group comprised professionals, people with visual impairments with and without mental health problems, and relatives of clients with visual impairments who had mental health problems. For professionals, the inclusion criteria were experienced in administering treatment and support to persons with a visual impairment and working at one of the two participating national organizations that provide support and services to persons with a visual impairment in the Netherlands. For experts by experience, the inclusion criteria were adults from the age of 18 years with a visual impairment and normal intelligence. Mental health problems were neither an inclusion nor exclusion criterion. The inclusion criterion for relatives from the age of 18 years and related to an adult with a visual impairment and normal intelligence is defined as an IQ greater than 90. Potential participants were identified through the assessment and treatment departments of the two participating organizations and were approached by the first author *via* email. Participating professionals were then encouraged to identify clients and relatives through a snowballing method.

### 2.3. Delphi round 1: Semi-structured interviews

Semi-structured interviews were conducted using an interview checklist developed by the first author. The questions were intentionally broad to ensure that the consensus could include as many experts’ opinions as possible. The checklist was reviewed by the research team—which included a person with a visual impairment; experts in the field of visual impairment and mental health disorders; and the authors—and subsequently categorized into seven main areas: visual impairment, emotions, stress, mentalization, multiple psychological problems, visual impairment and treatment, and collaboration. Table 1 presents examples of questions. In 2019 and 2022, the first author conducted interviews, which lasted a minimum of 15 min and a maximum of 140 min. All interviews were conducted at a place convenient for the participants and were voice-recorded. The audio files were transcribed verbatim by student researchers and then checked by the first author. The transcripts were numbered and did not contain directly identifiable personal information.

**Table 1:** Example of interview questions.

Questions—version clients and relatives
1. How do you show your emotion?
2. How do you recognize emotions in others?
3. Do you find it difficult to understand the feelings of others?
4. When do you experience stress?
Questions—version professionals
1. Can you elaborate on the theme ‘mentalization’?
2. How do you consider the client’s visual impairment in treatment?
3. What differences can be observed between someone who has a visual impairment from birth and someone who developed a visual impairment later in life.
4. Which adjustments are needed in the treatment for clients with a visual impairment?

### 2.4. Analysis of Delphi round 1

We applied reflexive thematic analysis informed by Braun and Clarke (2006, 2016, 2019). Reflexive thematic analysis is a method for identifying, analyzing, and reporting themes within data (Braun and Clarke, 2006). It is an appropriate method to examine for examining participants’ experiences (Braun and Clarke, 2006, 2016, 2019). The researchers (first author and independent researcher) first familiarized themselves with the data by actively reading the transcripts. The transcripts were then read again and preliminary ideas were noted. Then they used the software program “Atlas.ti” to independently conduct general coding by selecting the areas of text assigned to each respective code. In the next phase, the coded data were reviewed and analyzed to determine how the different codes could be combined on shared meanings to form themes and subthemes. We then revised and/or removed codes and (sub) themes to allow the most meaningful interpretation of the data. The first author documented how and why various codes and subthemes were merged or discarded for review by the independent researcher; any disagreements were resolved by further discussion.

Several steps were taken to ensure the quality of our study. First, we engaged in researcher triangulation: The first author and independent researcher independently coded 10 transcripts. We did not calculate the intercoder reliability but rather discussed the differences and similarities of our findings until reaching a consensus. Second, we adhered to the 15-point checklist criteria for good thematic analysis (Braun and Clarke, 2006). The criteria in this checklist cover the processes of transcription, coding, and analysis, as well as the overall process (e.g., the time spent on all phases of analyses and the creation of the written report).

### 2.5. Delphi round 2

Based on the concepts identified in round 1, a survey was developed and administered online using the Qualtrics software program. The participants were each sent an individualized email with a hyperlink providing access to the online questionnaire, or a printed copy of the questionnaire. Participants were sent a maximum of two reminders to complete the survey over the 4-week study period. In the survey, participants were asked whether they agreed or disagreed with the description of the key concepts (1 = agree and 2 = disagree). The consensus was defined as 70% of participants agreeing or disagreeing with the description. In a created text box, participants could also suggest an adjustment to the descriptions.

### 2.6. Analysis of Delphi round 2

Data were downloaded from Qualtrics into SPSS for Mac. Descriptive statics were used to describe participants’ characteristics and analyze the similarities. Qualitative data were imported into Microsoft Word and analyzed according to the open, axial, and selective coding procedure, as described for the analysis of Delphi round 1.

### 2.7. Ethical considerations

This study was conducted according to the guidelines of the Declaration of Helsinki and was approved by the ethical team of Bartiméus on 27 November 2018. All potential participants were provided with verbal and written information about the study. Written informed consent was obtained before the start of the interviews. After informed consent was obtained, all participants were numbered. All data were saved on a password-protected server.

## 3. Results

### 3.1. Participants

A total of 36 persons (25 professionals, 10 clients, and one relative) participated in round 1. Of these 36 participants, 22 (61.1%) completed round 2. Table 2 presents the characteristics of participants in round 1. Among the experts by experience, the age ranged from 18 to 70+ years, with a higher percentage being 40–55 years old. Among the professionals, the majority had worked with people with visual impairments for over 5 years. Most professionals were women. Among the experts by experience, the gender distribution was equal.

**Table 2:** Participants characteristics.

Participants	Variable	*N*	%
Clients with a visual impairment		10	
	Age		
	- 18–25	2	20%
	- 25–40	1	10%
	- 40–55	5	50%
	- 55–70	1	10%
	- 70–and older	1	10%
	Gender		
	- Male	5	50%
	- Female	5	50%
	Visual impairment		
	- From birth	5	50%
	- Later in life	5	50%
	Psychological problems	6	60%
	Psychological supports	9	90%
Professionals		25	
	Role		
	- Social worker	2	8%
	- Psychiatrist	1	4%
	- Psychologists	6	24%
	- Behavioral expert	7	28%
	- Team leaders	2	8%
	- Ambulant caregiver	4	16%
	- Caregiver	1	4%
	- Physician	1	4%
	- Ophthalmologist	1	4%
	Gender		
	- Male	4	16%
	- Female	21	84%
	Years of work experience		
	- 0–5	7	28%
	- 5–10	6	24%
	- 10–15	4	16%
	- 15–20	4	16%
	- 20 – and more	4	16%
Relative		1	
	Age		
	- 55–70	1	100%
	Gender		
	- Male	1	100%
	Visual impairment relative		
	- Later in life	1	100%

### 3.2. Findings

**Figure 1:**
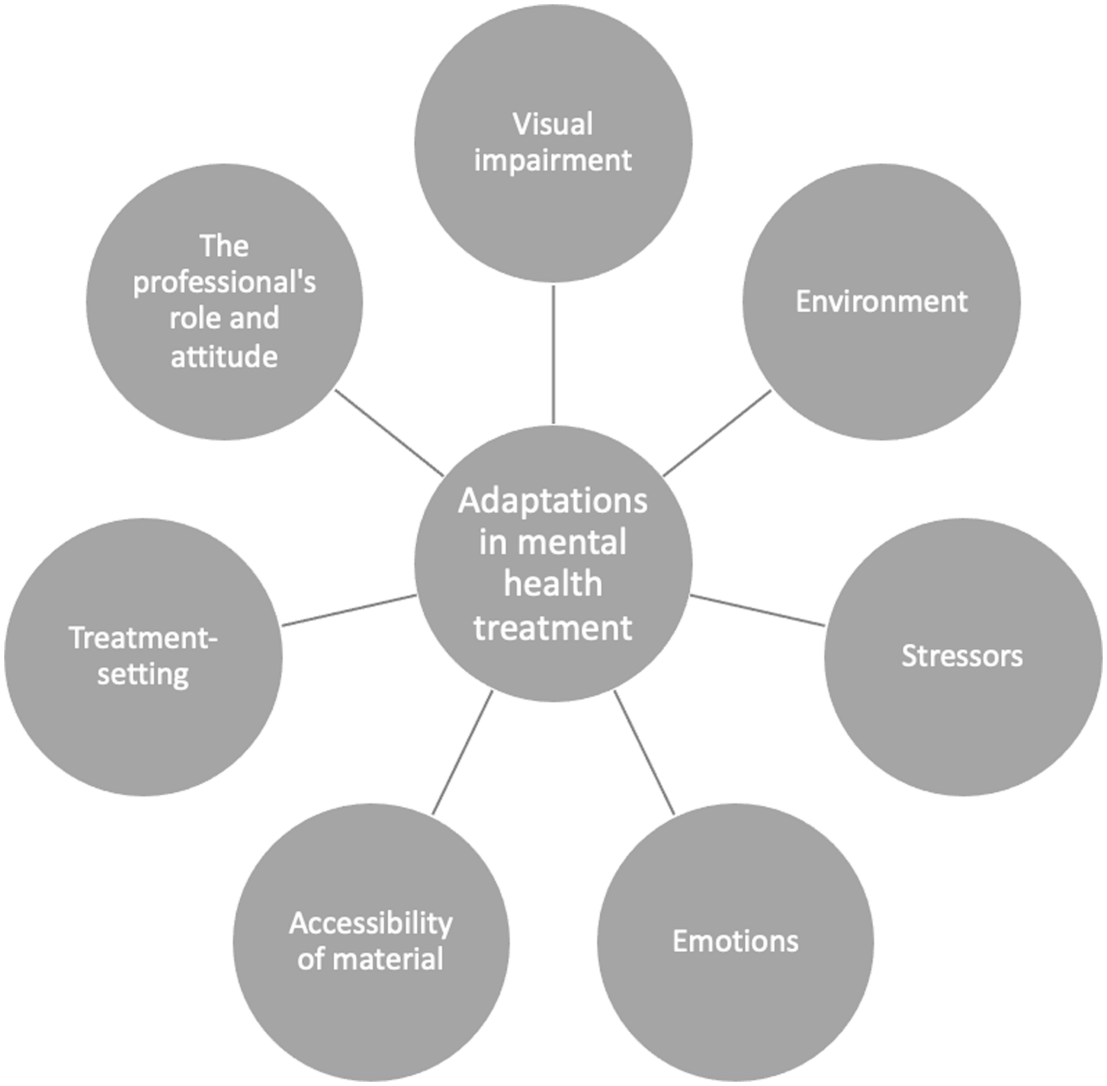
Overview of the categories related to the needed required adaptations in mental health treatment.

The Delphi-consultation resulted in the identification of seven categories or factors and related themes that were deemed important in the treatment of mental health problems among clients with a visual impairment. These categories were as follows: the visual impairment, environment, stressors, emotions, treatment-setting, the professional’s role and attitude, and accessibility of materials. These categories constitute a dynamic model (Figure 1).

### 3.3. Visual impairment

Several factors related to visual impairment affect mental health treatment (refer to Figure 1). These include the severity of the visual impairment; fatigue and limited energy; when the visual impairment began in their client’s lifespan; and having to cope with missing visual information, such as non-verbal signals and things that happen in the environment. Persons with a visual impairment are more likely to suffer from fatigue and limited energy, which affects their ability can cope with various aspects of the treatment process.

Several things cost extra energy. Going somewhere as a partially sighted or blind person can require a lot of energy. And when one has therapy three times a week, it takes a lot of extra energy. (Expert by experience 01).

I still sometimes notice that appointments are being cancelled, everything just takes a lot of energy. (Professional 01).

Another important factor is the point in the lifetime at which the client became visually impaired. Some clients who have a visual impairment from birth show stereotypical behavior, including repetitive movements that do not seem to have a clear purpose. These movements can confuse others and make social contact difficult.

Those who are blind from birth do have these, sort of tics it seems. Certain movements, for example wobbling slightly or rocking their heads. (Professional 03).

Clients with late onset visual impairment experience more loss processing than those who have visual impairment from birth. They are confronted with the loss of their vision on a daily basis.

The moment you suffer a loss of function during your adult life, such as becoming severely visually impaired because your retina is detached or some other condition that affects your eyes, you take that loss with you throughout the rest of your life. Because in all stages of life, you are confronted with limitations of that moment in time that weren’t there before you had that retinal detachment, for example. (Professional 04).

… When I heard that I was no longer allowed to drive, and that it was not responsible for me to do so—when I was still working, as I did continue to work for a long time—and then I drove my car to work on the last day. I then drove so fast that I got a great fine; I was so angry and sad. (Expert by experience 02)

Clients with a visual impairment from birth don’t know any better, and have to partially deal with the problems of processing in a different way. With them, they have to come to realize: “things are different in my life compared to those of others”. (Professional 05)

### 3.4. Social environment

It is considered important to involve the social environment in the treatment. Persons with a visual impairment depend on relatives for practical help and social–emotional support. When one has a partner with a visual impairment, many things are automatically arranged by the partner, such as transportation and housework. This can become a burden. The relatives also must deal with grief and loss themselves when their partner becomes visually impaired over time.

The client’s relatives are extremely important. The way they deal with the complaint is very important. When one has a partner who has a visual impairment, or is blind, the partner automatically arranges many things. He is then burdened more. And the grief, they too have to deal with grief and loss as their partner or child can no longer see. Relatives play an important role in the treatment; we actively try to involve them. (Professional 04)

Relatives might suffer when their partner gradually can see less. Therefore, it is best to include the client’s social environment in the mental health treatment and to also focus on how the treatment affects the relatives’ lives. During treatment, it is important to pay attention to both the client and the client’s loved ones, and also to the relationship between them. Psychoeducation for both the client and the people closest to them will support the client individually and their relationship.

I find it difficult, because my partner cannot do this or that any longer. (Relative)

*… During psycho-education, explain to family members what a visual impairment entails, and the consequences in daily life. And also, that the visual impairment can reinforce psychological problems. (Professional 06)*

### 3.5. Stressors

Visually impaired clients generally experience more stress compared to people without a visual impairment. Causes of higher stress levels may include feelings of powerlessness, fatigue, traveling, dependence, and coping with loss. In the treatment, it is important to identify the causes of stress.

*Usually, first look at what causes the stress. What are the stressors? Someone can set the bar very high and become totally stressed out because he has to do something. Articulating such things into words can support looking at what is possible. (Professional 07)*

*… I can get stressed just from cooking. First, I have to go shopping, oh I have to go into town, I’ll have a sandwich. But then I won’t do that and subsequently go into town not feeling at peace, and that experience is stressful for me. Or if someone asks me to do something that I cannot manage to do, I get completely stressed out. (Expert by experience 01)*

Many daily tasks require more energy for a client with a visual impairment and, therefore, stress can quickly build up. Visually impaired clients sometimes tend to overburden themselves—for example, when they are actually tired but still think they have to do the same amount of things as others in their surroundings. Again, psycho-education can be supportive.

*… it can be nice for them to hear that it is quite normal that if you are blind, things take more energy, and that this is the reason many blind people do not work 36 or 40 hours, because it is too strenuous, since you also have to travel, that it is different. (Professional 06)*

### 3.6. Emotions

The extent to which the client perceives and expresses emotions depends on a number of factors, such as the client’s developmental stage, personality, and environment. The client’s trust in the professional is important when discussing emotions during treatment.

*I hardly or do not express my emotions towards other people. This is because we didn’t really do so at home. You did not show emotions; that is the culture in which I was brought up. (Expert by experience 03)*

*I think this is achieved by first building up a relationship of trust, which creates space for this [talking about emotions like grief] to develop. (Professional 08)*

Assessing another person’s intentions is sometimes difficult for the client because the client cannot see the other person’s facial expression. However, it should be noted that they can detect other person’s emotions through their speech, intonation, and the person’s posture. Therefore, it is important for the professional (and any group member) to be aware of their own voice, intonation, and breathing.

*You can often tell from the voice whether someone is in a good mood or not.*

*Sometimes I can hear it in the way people breathe. (Expert by experience 06)*

Clients are not always aware of what they express non-verbally. For example, a client may express an emotion that does not match their actual feelings. During treatment, the professional should focus on the client’s non-verbal signals.

*What I often see is that people’s faces express a different emotion from what they are saying. I then articulate it into words—for example, “You say that you are not really bothered by it; however, you look quite sad” or “You say you are angry, but your voice sounds very cheerful”. (Professional 09)*

### 3.7. The professional’s role and attitude

The professional is a translator of what the client visually misses out on. In addition to translating non-verbal signals, it is also important for the practitioner to translate, for example, the silences that occur in a (group) conversation. In a conversation with several participants, clients with visual impairment may feel that they are not seen or heard when there are silences because they lack visual information and cannot see that someone is just thinking.

*You, as a professional, are aware of the fact that you are, or should be, the eyes of the client, and that you are helping him to understand the environment. (Professional 02)*

For clients, it is essential that professionals understand visual impairment as well as the influence it has on the client’s quality of life and psychological problems, and the extent to which psychological problems can affect visual impairment. This does not automatically mean that the professional will know or decide what the client can or cannot do. Clients can indicate what they can or cannot do with regard to their visual impairment. The professional and the client should agree on what the client likes and dislikes in terms of support, and what the client needs from the professional to feel comfortable.

*The fact that you have knowledge of the influence that a visual disability can have on your development, on your well-being, on your quality of life, and on your depression or anxiety. That can also offer some recognition. (Professional 05)*

*Which options are available to the client to compensate for his visual impairment, and how can the client use those options? And I think that I always do my best to ask the client how he wants me to act in such situations. This can be a small thing, when I go out to pick someone up from the waiting room, I ask “How can I best support you?” (Professional 05)*

Participants indicate that the professional should be concrete and transparent. For all clients, but especially for clients who have never been able to see, one should more specifically describe expressions and abstract things and be transparent about how the professional feels that day. For example, a bad day certainly influences the professionals’ attitudes and reactions, and visually impaired clients have developed a kind of sensitivity to this.

*… it is nice if someone takes into account that you cannot see. So, for example, with descriptions, or pointing out where something is—articulating where it is into words. Or articulating where you need to go into words. And don’t use “here”, “there”, or any words like that. (Expert by experience 02)*

*You can easily feel if someone is not feeling well. The other person does not even have to say anything; I can just feel it. (Expert by experience 03)*

*Most clients know very well when you are not feeling well. Therefore, is it important that you yourself articulate it into words—for example, “I have a cold, I can’t handle as much today”, or if something touches you, that you say “Gosh, I notice that this really touches me”. (Professional 09)*

### 3.8. Treatment setting

The setting in which treatment is provided is an important factor for clients with visual impairments. The treatment location should be easily accessible for the client. When this is not the case, the professional should make arrangements to provide treatment in a more suitable location. Upon securing an accessible treatment location, it is also important to consider that the client may not be able to see the setting of the location. A description of the room will be needed—including how it is situated and whether people can see you when they pass by.

*If I were to invite someone here who is visually impaired, I would ask “Is there anything I can do for you?”, for example, “can I pick you up at the bus stop?” This would be specifically related to the accessibility of the appointment location. (Professional 11)*

*When clients enter the room, you articulate what it looks like in words—where someone might want to sit, what someone finds comfortable … If someone cannot see the setting of the room, you articulate it into words for them. (Professional 13)*

Other factors include the light in the room and environmental sounds. Clients may be inconvenienced by the light in the treatment room; this is something that the professional should ask the client. In a group meeting, it is important to check with all the participants beforehand whether the light in the room is comfortable for them. With regard to environmental sounds, clients with a visual impairment often tend to be auditorily attuned and, thus, sensitive to environmental sounds. Therefore, it is important that the professional pay attention to environmental sounds.

*You check beforehand where to position someone, when also considering the light in the room. You should not place someone who is visually impaired facing the light. (Professional 12)*

*I pay attention to what I hear, because someone is of course mainly auditorily attuned when that is still intact. Environmental sounds can be very disturbing, so I ensure minimal noise. (Professional 13)*

In the case of group treatments, it is important to consider group composition. It is preferable not to put visually impaired and blind people in the same group because they encounter different types of problems.

*I do not like to be in a group with visually impaired people; I find it annoying and confronting. For example, someone once said “Now I can’t ride a bike any longer”. While I haven’t been able to do that for a very long time now because I can’t see anything at all. At such moments, I find that they are complaining and should be happy with what they can still see. That is why a group with blind and visually impaired people combined does not work. (Expert by experience 03)*

*A mixed discussion group of people who are blind and people who have residual vision does not work. People who have residual vision have issues with what they can no longer see. And someone who is blind says “I would do anything to see what you are still able to see”. That is very painful. (Professional 14)*

### 3.9. Accessibility of material

Materials used in the treatment must be accessible to the client. The materials that work best will vary depending on the client. A client may prefer for information to be shared auditorily, digitally, or *via* braille. To support this, it is important for the professional to find out what type of reading materials the client uses and what they can and cannot see.

*I would first ask what kind of reading materials someone uses. I ask what he can and cannot see. Someone who is visually impaired can often still see things in a diagram. A blind client will not be able to see a diagram. Then you can explain things, or you can make it tangible. I evaluate the needs per client. (Professional 07)*

The way in which a client can do homework assignments will also depend on the client. Whereas a non-visually impaired client can write down his homework, a visually impaired client may prefer to record a homework assignment. A professional should work with visually impaired clients to look for options that are feasible for them.

*… If I give a homework assignment, he often speaks the homework into a recorder and then he listens back. But also, the other way around—for example, if you give a relaxation exercise, we often record it together on the telephone or tape recorder, so that the client can listen to it at home. (Professional 01)*

When the client can still see and read a little, it is often advisable to enlarge the text or questionnaires or to use an extra-large font. It is also possible to work with contrasting colors or, when the professional wants to draw something, to use a thick black marker on a whiteboard or paper.

*They do offer the possibility to enlarge the questionnaires, or to read it out loud, for example. (Client 07)*

## 4. Discussion

In this qualitative study, we established consensus opinions on a range of topics relevant to the adjustment of mental health treatments for people with visual impairments. This study examined the personal experiences of clients, relatives, and professionals regarding the adaptations that are required in psychological treatments for people with visual impairments. We identified seven factors that should be attended to, depending on the individual needs: visual impairment, social environment, stressors, emotions, the professional’s role and attitude, treatment setting, and accessibility of materials. Our findings may help mental healthcare providers in low-vision service organizations and regular mental healthcare organizations to understand the needs of visually impaired clients with psychological problems and to adjust the psychological treatment accordingly.

Acknowledgment of visual impairment plays a role. For example, a client with a visual impairment can be recurrently confronted with their loss as new problems or situations repeatedly redefine their loss. Professionals should be aware of the reoccurring confrontations with vision loss, which may lead to the worsening of psychological problems. The participants mentioned the important role of the time point at which their visual impairment arose. Compared to clients with a visual impairment from birth, clients with late-onset visual impairment experience more problems with vision loss as new situations and new problems keep reminding them of their loss—for example, no longer being able to cycle can result in another confrontation with being visually impaired. Professionals should be aware of such reoccurring confrontations with visual impairment.

Clients often experience practical and emotional support from their social environment. It should be noted that the relatives of visually impaired clients can also struggle with the client’s vision loss. Vision loss may lead to changes in roles and responsibilities among the relatives and place a burden on the relatives. The literature shows that a disabled partner’s increased need for support—such as emotional support or help with daily activities—can lead to a lack of reciprocity in the relationship, which can create a source of conflict between the client and their relatives (Bernbaum et al., 1993; Melton et al., 1995; Waern et al., 2002; Williams, 2002; Cimarolli et al., 2004; Enoch et al., 2022). Providing psycho-education to both clients and their relatives could facilitate a better understanding of specific types of vision loss and their differential effects on daily functioning. An evaluation of a psycho-educational support group for partners of adults with visual impairment demonstrated several changes after participation, including increased knowledge about what the partner with a visual impairment can see and do and improved communication between the partner and the client (Cimarolli et al., 2004; Cimarolly and Boerner, 2005). Based on this study, psycho-education for partners should include knowledge of the partner’s visual impairment, learning from others how to adapt to the situation and how to cope with different problems, information regarding emotional issues, and information about managing change and dealing with stress.

Participants mentioned that adults with visual impairments needed to receive information from professionals about their visual impairment, the impact of visual impairment on mental health problems, and the extent to which mental health problems can affect visual impairment. In another qualitative study, clients emphasized the need for the professional to provide information about the impact of visual impairment on mental health problems (van Munster et al., 2021). However, different studies have shown that professionals provide insufficient knowledge about how visual impairment influences mental health problems (Rees et al., 2009; Harrisson et al., 2010; Nollett et al., 2019). It is important that professionals educate clients with visual impairments about how their impairment impacts both activities of daily life and mental health problems.

Various studies show that people with visual impairments experience barriers to obtaining information because it is inaccessible. For example, flyers including important information about treatment, and information provided on a poster in a waiting room, are so vision-focused that they cannot be accessed by persons with visual impairments (Harrisson et al., 2010; van Munster et al., 2021; Warren et al., 2016). The participants in our study emphasized the importance of using accessible and tailored information materials, for example, audio recordings or enlarged texts.

Participants in our study also mentioned that in the case of group treatments, it is important to consider group composition. Participants advised having different groups for blind versus visually impaired clients. As group treatments are not common in the field of treating persons with a visual impairment, future research should more specifically look into group composition—for example, evaluating a group composition in a treatment with a structured group protocol. In addition, the type of group intervention may play a role in the group composition.

To the best of our knowledge, this study is the first to explore which factors are important for the adaptation of psychological therapies from the perspective of visually impaired adults, professionals, and relatives of adults with a visual impairment. The qualitative design allowed us to understand the actual experiences of this expert group when discussing this topic. Our study included a heterogeneous group, with professionals having different work experiences and clients with different genders, ophthalmic diagnoses, and age groups.

The range of themes and factors could have been more exhaustive, or even narrower if the expert’s group had a different composition. All experts were linked to a low-vision service organization ([BLINDED FOR REVIEW]). One professional worked at both a low-vision service organization and a regular mental healthcare organization. Including professionals who do not work with this client population on a daily basis could lead to a different picture of what adaptations are needed in the psychological treatment of clients with vision impairment.

We could include one relative in this study. This may be due to the burden experienced by many relatives. Our study participants in the study mentioned that many things are automatically arranged by the partner such as transportation and housework. Different studies show that clients with a visual impairment may need greater assistance in activities of everyday living, which has both an immediate and lasting impact on relatives (Glass and Maddox, 1992; Palmer and Glass, 2003; Bambara et al., 2009). Relatives may be challenged—for example, in finding the right balance between seeking independence from their partner and providing assistance. Future research may include conducting a comprehensive study on the relatives’ experiences in general and, more specifically, on their role, wishes, and needs during psychological therapy for their partner with a visual impairment.

In Delphi studies, a third round is often conducted, in which the experts are asked to re-assess statements that did not reach consensus to ensure that they are given due consideration before they are discarded. However, in our present study, the consensus on the responses was reached in round 2, thus, there was no need to conduct round 3. The themes were recognized and confirmed, which increases the validity of this study.

It is important that professionals are aware of the factors that were deemed important in the mental health treatment of clients with visual impairments. Factors regarding clients’ visual impairments, such as the severity of the impairment, influence the extent to which adjustments must be made in psychological therapy. The role and attitude of the professional is the most important factor in treatment. Professionals are expected to have sufficient knowledge of visual impairment and how visual impairment impacts psychological problems. Moreover, the professional is a translator of what the client visually misses. In practice, specific treatment adjustments are made for each individual client. Overall, treatment materials should be accessible to all, and the role of relatives should be addressed during therapy. Finally, it is important to perform a continuous evaluation of the treatment and to fine-tune the method to the client’s specific needs.

## Data availability statement

The original contributions presented in the study are included in the article/supplementary material, further inquiries can be directed to the corresponding author.

## Ethics statement

The studies involving human participants were reviewed and approved by the ethical team of Bartimeus was on 27-11-2018. The patients/participants provided their written informed consent to participate in this study.

## Author contributions

JB coordinated and performed the study, wrote the first version and the revisions of the manuscript. PS provided supervision and contributed to the manuscript and the revisions. Both authors finalized and approved the submitted version.

## Funding

This research is funded by the Netherlands Organization for Health Research and Development ZonMW, the Netherland, project number: 6390 10004. The funding organization had no involvement in study design, data collection, data analysis, manuscript preparation and publication decisions.

## Conflict of interest

The authors declare that the research was conducted in the absence of any commercial or financial relationships that could be construed as a potential conflict of interest.

## Publisher’s note

All claims expressed in this article are solely those of the authors and do not necessarily represent those of their affiliated organizations, or those of the publisher, the editors and the reviewers. Any product that may be evaluated in this article, or claim that may be made by its manufacturer, is not guaranteed or endorsed by the publisher.
